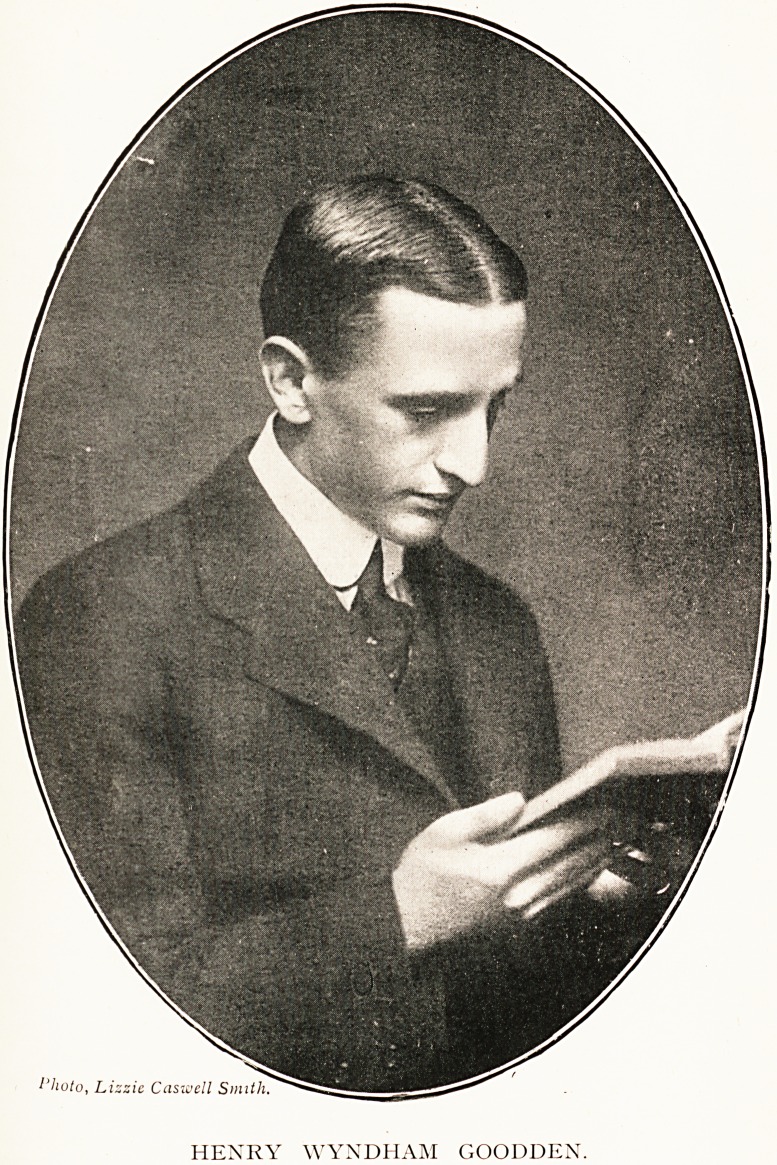# Henry Wyndham Goodden

**Published:** 1915-06

**Authors:** 


### Obituary.

### HENRY WYNDHAM GOODDEN, LIEUT. R.A.M.C.

THE news that Henry Goodden had been killed in action near Ypres brought sad regrets to many hearts in Bristol, especially to those who had been his contemporaries and associates at the Royal Infirmary. Owing to his prolonged period of medical study Goodden could claim more than the usual number of school contemporaries. As he pursued his curriculum, passing from Bristol to Vienna, from Vienna to Paris, and in the intervals, as well as at the end, returning to his Alma Mater, Goodden was the cherished friend and enlivening spirit of successive generations of students.

His manner of studying medicine was stamped with an originality all his own. In no great hurry to acquire a conventional diploma qualifying him to practise, he was an omnivorous learner. By no means a mere memoriser of other men's facts and formulas, he went quietly and leisurely through the pastures of medicine, displaying from his earliest days a shrewd appreciation of values, digesting rapidly and completely, so that what he learned he knew, provided it was worth knowing or learning.

Goodden was born in 1883 at Swindon, Wilts. He was educated at Clifton College and the Bristol Medical School, subsequently at Vienna under Professors Eiselberg, Politzer, and others.

He continued his medical studies in Paris, where he was clinical clerk at the Hôtel Dieu, being the first English student to hold this appointment. He also worked at the Salpétrière Hospital. Unlike the majority of English students, he did not leave his foreign studies and travels to postgraduate days, but entered and attended the usual student curriculum. This he was enabled to do by his great gift of acquiring foreign languages, even Russian not coming amiss to him. After taking his degrees in the University of Bristol, Goodden held several resident posts at the Bristol Royal Infirmary, where the outbreak of the war found him Senior Resident Officer and House Surgeon. He promptly volunteered for service, and was gazetted Lieutenant (temporary) in the R.A.M.C., being attached to the Royal Irish Regiment.

He was present with his regiment at many fierce engagements, and was wounded by shrapnel during the battle of the Aisne, when his life was saved by a silver cigarette case in his left breast-pocket. On another occasion a bullet passed through the front of his cap without touching him.

On May 9th, 1915, he fell on the field of honour.

Colonel S. Moriarty, of the 2nd Battalion Royal Irish Rifles, wrote the following letter describing how bravely he met his death:—

DEAR MR. GOODDEN,

In reply to your letter of 13th, your son was killed north of Ypres. The circumstances were as follows. The battalion was in the trenches and suffering from fire, which was very heavy. In order to look after the men he came up with some shell dressings and did what he could. He left the trench then

*Photo, Lizzie Caswell Smith.*

HENRY WYNDHAM GOODDEN.

to go back. I endeavoured to dissuade him from doing so. On his way back he was hit I believe in three places, so the orderly who I sent with him states, by Maxim gun. He was buried by another regiment, but so far I have not got the exact spot, but am trying to get it for you. The erection now of a stone is quite impossible, but I am sure that the place is marked, and if I am able later on to do anything I will do so. It may sound as if no care had been taken, but it is a very dangerous spot either by day or night. I miss him more than I can say, and not only myself but we *all* do so. I have forwarded his name for a mention in dispatches, but of course the list is only a regimental one, and may not get through into the general list, as there will be so many names. You would probably like to see what form my recommendation took, so I send you a copy.

I was very fond of your son, and if I may say so, I am most deeply grieved at his death personally, and from a regimental point of view am certain I cannot have another who will in any way approach him from whatever point of view I may look.

I am sending some effects which I hope will arrive safely, and would be glad to hear if they do so.

Please accept all our most deep sympathy in your loss.

I am,
Yours sincerely,
S. MORIARTY.

One of Goodden's contemporaries at the Infirmary, Captain V. B. Green-Armytage, I.M.S., writing from "Somewhere in France," sends the following appreciation of the man as his intimate friends knew him :—

"As one of the Bristol men out here will you let me express through you to his School how deeply grieved I am at Henry Goodden's death. He and I were at Clifton together, and after that joined the old Medical School about the same time. Those were the old happy, haphazard days of the evil-smelling shove-halfpenny students' room, the dim and dingy dissecting-room, with its limpet-like kidneys on the ceiling, and the esoteric melancholy of droning lectures and labs., brought into happy relief by a vivid cricket or football match and its finale, or a Clubs' Union Meeting!

"Henry in those days was always Secretary of everything, and it would have been impossible to find a better. For nimble-witted and keen though he was, his post was no sinecure, it included everything, from buying the tickets to supplying the beer and bailing out delinquents on many an 'out' match, such as Bridgwater. Those were happy days, and Henry's cheeriness was always his dominant feature, as Pa Davis, Dick Vaughan, Bill Webb, and many another would testify. And then came the old B.R.I., with all its traditions and associations. The old students' room, the garden and the cottage, through all these and other influences Henry passed, picking his way slowly but surely, and losing nothing in knowledge of men and affairs. He had an originality and freedom of expression of his own, and in that he had studied and, above all, kept his eyes and ears open in Vienna and Paris, when at length he settled down and occupied the position of S.R.O. at the B.R.I. his future professional success was assured.

"It was a great pleasure to me, after three months at the front in France, to meet him one day quite casually in January. He was, I found, not the least changed—ever cheery and possessed, and full of his experiences from the time he left the B.R.I. early in August till December. In March and April we met quite frequently, and the last time I was having tea with him in his mess his C.O. spoke very highly of Henry, saying how nearly he had been captured and injured on the Aisne, and how glad he was still to have with them at least one of the original mess that left England in August. His regiment went up then to the Ypres sector, and although I was sent up at the end of April or early in May to lend a hand with the 'gas' and other casualties, I did not see him again. Both he and I had been there before, but this time the fighting has been even greater than in the autumn. I do not know how his end came, but knowing him as I do, and what he was, I am certain he died at his post fearlessly, encouraging and doing his utmost for all till the end. We have all of the old B.R.I. lost a great friend, and the profession a good man. Out here with his regiment and at home with you his name and what he stood for will be remembered long."

## Figures and Tables

**Figure f1:**